# A Plant Worthy of Further Study—Volatile and Non-Volatile Compounds of *Portenschlagiella ramosissima* (Port.) Tutin and Its Biological Activity

**DOI:** 10.3390/ph15121454

**Published:** 2022-11-23

**Authors:** Elma Vuko, Sanja Radman, Igor Jerković, Juraj Kamenjarin, Irena Vrkić, Željana Fredotović

**Affiliations:** 1 Faculty of Science, University of Split, Ruđera Boškovića 33, 21000 Split, Croatia; 2 Faculty of Chemistry and Technology, University of Split, R. Boškovića 33, 21000 Split, Croatia

**Keywords:** *Portenschlagiella ramosissima*, volatilome, essential oil, hydrosol, gas chromatography-mass spectrometry, headspace microextraction, myristicin, antiphytoviral, antiproliferative activity

## Abstract

New and detailed data are presented on the phytochemical composition of the volatile and non-volatile organic compounds of the Mediterranean endemic species *Portenschlagiella ramosissima* (Port.) Tutin. Both the essential oil and hydrosol were obtained from the air-dried plant by hydrodistillation and analyzed by gas chromatography-mass spectrometry. The volatile compounds from the fresh and air-dried plants and from the hydrosol were isolated for the first time by headspace solid-phase microextraction using two fibres of different polarity. The benzene derivative group was the predominant group in all samples, with myristicin being the most abundant component of all. The non-volatile compounds of the methanol extract were analyzed by ultra-high-performance liquid chromatography–high-resolution mass spectrometry with electrospray ionisation, and three flavonoid glycosides, one anthocyanidin glycoside, and lipid derivatives were detected. Both the chemical composition and biological activities of this plant have been described in a very limited number of publications, making it an interesting source for further study. The antiphytoviral activity of the essential oil and hydrosol showed that both extracts significantly reduced the number of lesions on the leaves of local host plants infected with tobacco mosaic virus. Moderate antiproliferative activity of the methanol extract was detected in three cancer cell lines, cervical cancer cell line, human colon cancer cell line and human osteosarcoma cell line, using the MTS-based cell proliferation assay. Based on the results, we highlight this plant as a new source of bioactive compounds and natural phytotherapeutic agent that deserves further investigation.

## 1. Introduction

The species *Portenschlagiella ramosissima* (Port.) Tutin belongs to the family Apiaceae whose members are cosmopolitan and distributed mainly in the northern temperate zone. They are mostly herbaceous plants, rarely shrubs, and only a few taxa develop as trees [1]. Due to the content of essential oils, members of the Apiaceae family have a specific odour and taste, are known for their medicinal properties and also used as spices. The subject of this study, *P. ramosissima*, a species endemic to the Mediterranean regions of Croatia, Bosnia and Herzegovina, Montenegro, Albania and Italy [2], grows in the fissures of limestone rocks. Only two papers describe the phytochemical composition of its volatiles, the first referring to a plant collected in Croatia and the second to a plant collected in Montenegro [3,4]. From the literature, it appears that this plant is a rich source of myristicin, although the literature data describing the essential oil differ in terms of the predominant component. In Montenegro, myristicin was the predominant compound, while Croatian *P. ramosissima* contained a high percentage of myristicin, although *γ*-terpinene was the most abundant component. In addition to myristicin and *γ*-terpinene, terpinolene, elemicin, sabinene, *α*-terpinene, and other essential oil constituents were among the more abundant components in the volatilome of *P. ramosissima* [4].

Since ancient times, essential oils have been used in traditional medicine around the world for their beneficial effects on human health. The described effects of essential oils, such as antiproliferative, antimicrobial, antioxidant, insecticidal and other effects [5,6,7], are constantly being supplemented by new findings and new effects such as antiphytoviral activity [8,9]. In addition to their role in folk medicine, essential oils are components of pharmaceuticals and are used in the agricultural and food industries. Numerous preclinical studies have documented various biological activities in a variety of cellular and animal models, also elucidating their mechanisms of action and pharmacological targets. Nevertheless, the paucity of human studies limits the potential of essential oils as effective and safe phytotherapeutics [10]. In an ecological context, secondary metabolites establish a relationship between plants and their ecosystem; for example, volatile compounds can repel phytophagous organisms, including viruses and phytoplasma vectors [11]. Among the non-volatile organic compounds of plants, there are also many bioactive constituents with numerous beneficial effects for plants and also great health benefits for humans. Therefore, some of them, such as flavonoid glycosides, are used as drugs and dietary supplements due to their good bioactivity and low toxicity [12]. The antiproliferative effect of methanol extract has already been demonstrated for some Apiaceae and other aromatic plants, but as far as we know, no such data have been published for *P. ramosissima*. Most of the studies performed so far on the antiproliferative and cytotoxic activity of species of the Apiaceae family have dealt mainly with essential oils [13,14,15,16,17,18]. Somewhat fewer studies have been conducted with various extracts of Apiaceae plants, including methanol, ethanol, dichloromethane, chloroform, and others [19,20,21,22,23].

Considering the insufficient data describing the phytochemical composition of *P. ramosissima*, the first objective of our research is a detailed analysis of the volatilome of this plant species. Volatile organic compounds (VOCs) were isolated during the hydrodistillation process in the hydrophobic fraction (essential oil) and in the hydrophilic fraction (hydrosol) and then analyzed by gas chromatography-mass spectrometry (GC-MS). For the first time, VOCs were also detected in the fresh and air-dried plant material by headspace solid-phase microextraction (HS-SPME) using two fibres with different polarity, and non-volatile compounds (NOCs) were analyzed by ultra-high-performance liquid chromatography–high-resolution mass spectrometry with electrospray ionisation (UHPLC-ESI–HRMS). The very limited research on the biological activities of *P. ramosissima* has led us to investigate the biological potential of its VOCs and NOCs to highlight the valuable natural compounds hidden in aromatic plants. In addition to essential oils, hydrosols, the by-products of water distillation, are an interesting source of bioactive compounds because they contain water-soluble components that are generally less concentrated than oils. Concerning the use of natural products as eco-friendly phytotherapeutics, the aim is to demonstrate the beneficial antiphytoviral and antiproliferative activities of biogenic VOCs and NOCs from aromatic plants. We considered it important to carry out research that would open up the possibility of using all the products of the distillation process according to green chemistry methods.

Overall, this study aims to provide a detailed analysis of the phytochemical composition of the plant material and the volatile and non-volatile extracts of *P. ramosissima*, which, complemented by the biological activities, will reveal a possible use of this plant as a new source of bioactive constituents and as a natural phytotherapeutic agent.

## 2. Results and Discussion

### 2.1. Composition of Volatile Organic Compounds Obtained by Hydrodistillation—Essential Oil and Hydrosol

**Figure 1:**
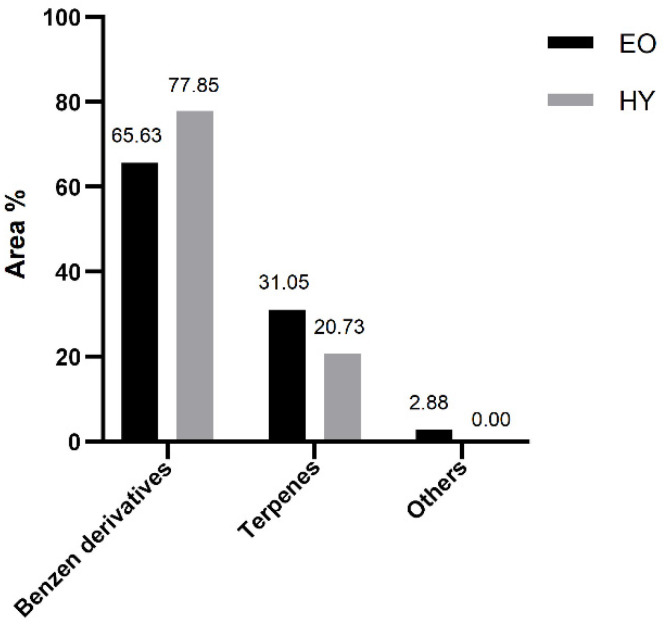
Volatile organic compounds of air-dried *P. ramosissima* isolated by hydrodistillation, analyzed by gas chromatography-mass spectrometry and sorted by structural groups. EO—essential oil; HY—hydrosol.

**Figure 2:**
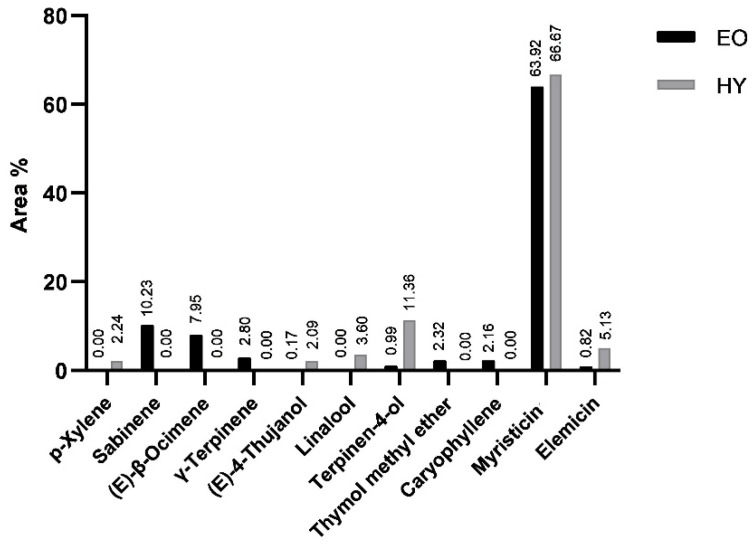
The most abundant compounds in air-dried *P. ramosissima* samples obtained by hydrodistillation and analyzed by gas chromatography–mass spectrometry. EO—essential oil; HY—hydrosol.

During hydrodistillation of the air-dried plant, both the essential oil (EO) and the hydrosol (HY) were isolated and analyzed. The percentage of compounds identified was 99.56% (EO) and 99.48% (HY) of the total compounds detected (Table 1). In both samples, the group of benzene derivatives (Figure 1) dominated with myristicin as the most abundant constituent (63.92%, EO; 66.67%, HY) (Figure 2). Its isomer (*E*)-isomyristicin was detected in a small amount in EO (0.04%), but not in HY. Elemicin (0.82%, EO; 5.13%, HY) and its isomer, (*E*)-isoelemicin (0.01%, EO; 0.15%, HY), were also detected and identified.

Terpenes were the second most important group of identified compounds, with monterpenes being the most abundant (Figure 1). Hydrophobic terpenes dominated in EO, while terpene alcohols dominated in HY. The most abundant terpenes in the essential oil were monoterpenes sabinene (10.23%), (*E*)-β-ocimene (7.95%), γ-terpinene (2.80%), and aromatic monoterpene thymol methyl ether (2.32%). None of them was detected in the hydrosol. The mentioned compounds were detected in *P. ramoississima* in a previously published article [3,4] but in different proportions. In hydrosol, the monoterpene alcohols terpinen-4-ol (11.36%), linalool (3.60%), and (*E*)-4-thujanol (2.09%) dominated among terpenes. Monoterpenic alcohols were either not detected or their content was below 1.00% in the essential oil. In the group of other aliphatic compounds, the abundance was greater in EO than in HY (Figure 1). The differences in the composition of EO and HY are the result of extraction of different compounds’ polarity.

### 2.2. Headspace Composition of the Volatile Organic Compounds Isolated by HS-SPME—Plant and Hydrosol

**Figure 3:**
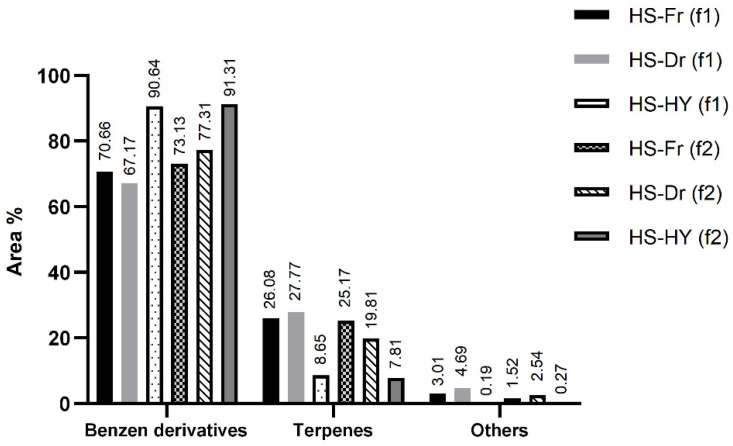
The volatile organic compounds of *P. ramosissima* extracted by headspace solid-phase microextraction, analyzed by gas chromatography-mass spectrometry and sorted by structural groups. Extraction by divinylbenzene/carboxene/polydimethylsiloxane fibre (f1): HS-Fr (f1)—fresh *P. ramosissima*; HS-Dr (f1)—air-dried *P. ramosissima*; HS-HY (f1)—hydrosol of air-dried *P. ramosissima*. Extraction by polydimethylsiloxane/divinylbenzene fibre (f2): HS-Fr (f2)—fresh *P. ramosissima*; HS-Dr (f2)—air-dried *P. ramosissima*; HS-HY (f2)—hydrosol of air-dried *P. ramosissima*.

**Figure 4:**
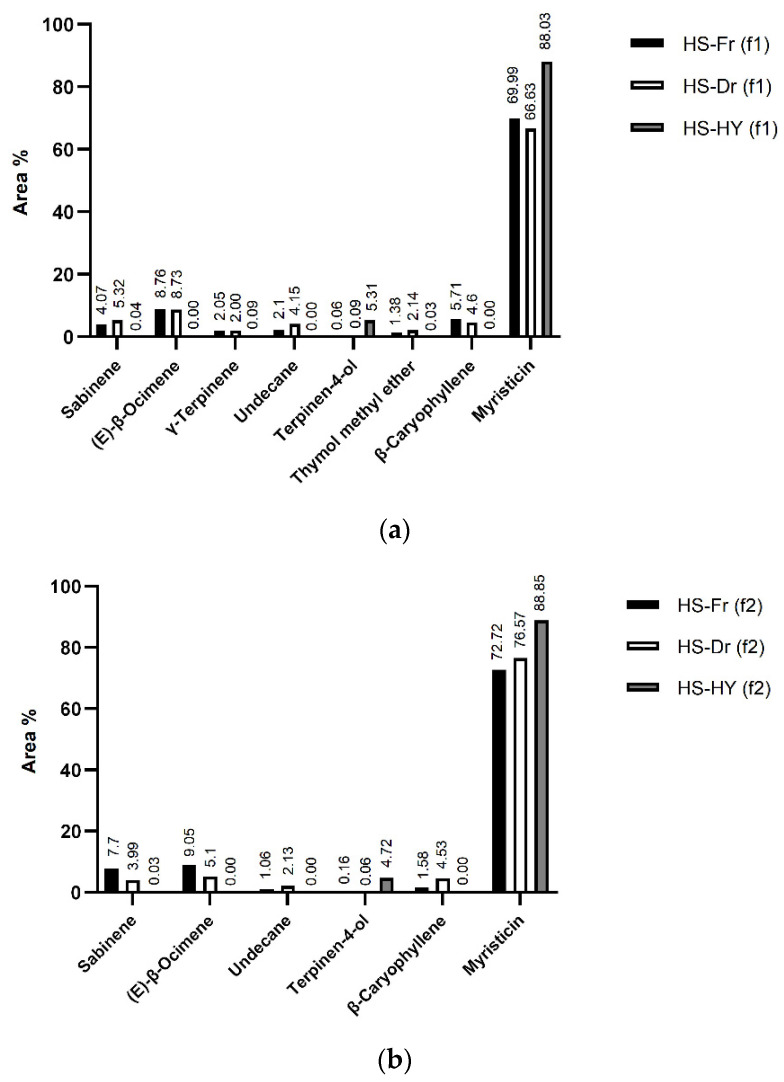
The most abundant compounds in the headspace composition (HS) of fresh (Fr) and air-dried (Dr) plant material and hydrosol (HY) on (**a**) divinylbenzene/carboxene/polydimethylsiloxane fibre (f1) and (**b**) polydimethylsiloxane/divinylbenzene fibre (f2).

For the first time, both fresh (HS-Fr) and air-dried (HS-Dr) plants as well as hydrosol obtained from hydrodistillation (HS-HY) of the air-dried plants were isolated by HS-SPME. To obtain more accurate information on the headspace composition, two fibres with different polarity were used: divinylbenzene/carboxene/polydimethylsiloxane (DVB/CAR/PDMS, f1) and polydimethylsiloxane/divinylbenzene (PDMS/DVB, f2). In HS-Fr, 99.75% (f1) and 99.82% (f2), in Hs-Dr 99.63% (f1) and 99.66% (f2), and in HS-HY 99.48% (f1) and 99.38% (f2) of the total VOCs detected were identified. The identified compounds were classified into three structural groups: benzene derivatives, terpenes, and others (Figure 3). The benzene derivative group was the dominant group extracted from both fibres in all samples: HS-Fr (70.66%, f1; 73.13%, f2); HS-Dr (67.17%, f1; 77.31%, f2), and HS-HY (90.64%, f1; 91.31%, f2) with myristicin as the most abundant component of all (Table 2). The second most abundant group of compounds, terpenes, differed from fibre to fibre. When analyzed with DVB/CAR/PDMS (f1), the fibres HS-Fr (26.08%) and HS-Dr (27.77%) had a similar percentage of identified terpenes, while analysis of the dry plant material with PDMS/DVB (f2) noted the decreased percentage of terpenes (25.17%, HS-Fr; 19.81%, HS-Dr). Although it could be concluded that some of the terpenes were lost by drying, analysis with f1 fibre showed a similar percentage of identified terpenes in fresh and dry material. This comprehensive analysis of fresh and dry plant material using two fibres for the first time thus gives a complete insight into the composition of volatiles of this plant species. Monoterpenes are again prevalent, with (*E*)-β-ocimene, sabinene, γ-terpinene, and thymol methyl ether being the most abundant (Figure 4). The proportion of sesquiterpenes in the headspace of fresh and air-dry plants is greater than in hydrosol, with β-caryophyllene being the most abundant. Terpene alcohols were present in greater abundance in HS-HY than in HS-Fr and HS-Dr. Terpinen-4-ol was the one with the largest percentage (5.31%, f1; 4.72%, f2) (Figure 4). Among others, aliphatic compounds were the most abundant especially in the headspace of the air-dried plant. This could be the result of fatty acid degradation. The phytochemical composition of the fresh and dry material as well as the isolated extracts is important for further studies and possible applications of this plant in daily life. The analyses performed revealed a fairly constant composition of the volatile components, indicating that both fresh and dry plant material can be used.

### 2.3. Non-Target Screening of Non-Volatile Compounds in Methanol Extract

The dried plant was extracted with methanol and analyzed by UHPLC-ESI–HRMS. Both positive and negative modes were recorded. The probable and possible structure and/or class identification of the major compounds in terms of signal intensity in positive electrospray mode were based on their elemental composition and MS/MS spectra (Table 3). Three flavonoid glycosides, one anthocyanidin glycoside, and lipid derivatives were found. The most abundant compound was sphingolipid hexadecasphinganine, compound 4 with the molecular structure C_16_H_35_NO_2_ (Figure S4). Sphingolipids as essential bioactive cell components play an important role in miscellaneous parts of plant development as well as environmental response. Inducing programmed cell death, they participated in defence against bacteria and fungi pathogens [24]. Fatty acid amide erucamide (compound 6, C_22_H_43_NO) was the second most abundant compound (Figure S6). Erucamide participates in strong plant–microbe interactions [25]. Another compound (compound 5, C_18_H_28_O_4_) with a role in the defence reactions has been found and belongs to the class of octadecanoids (Figure S5). One glycerophosphoserine (compound 8, C_37_H_74_NO_9_P) was identified as either 1-octadecyl-2-tridecanoyl-glycero-3-phosphoserine (PS (O-18:0/13:0)) or 1-hexadecyl-2-pentadecanoyl-glycero-3-phosphoserine (PS (O-16:0/15:0)) (Figure S8). Compounds 1 (C_21_H_20_O_12_), 2 (C_21_H_20_O_11_) and 3 (C_22_H_22_O_11_) were classified as flavonoid C-glycosides containing hexose ring. By searching the MassBank database, the selection of possible candidates was narrowed down; compound 1 was one of following isomers: isoquercitrin, hyperoside, quercetin or spiraeoside; compound 2 was one of flavone C-glycoside; compound 3 was one of flavone C-glycoside (Figures S1–S3). In plants, they are involved in the defence system, especially against UV radiation [26]. Regarding anthocyanidin glycoside (compound 7, C_28_H_33_O_15_), it was identified either as peonidin 3-rutinoside or peonidin 3-rhamnoside 5-glucoside (Figure S7). The anticancer activity of the above compounds has already been demonstrated in numerous studies [27,28,29].

The chromatograms obtained in the negative electrospray ionization mode gave much less intense peaks and much more intense background and were therefore not analyzed.

### 2.4. Antiproliferative Activity of Hydrosol and Methanol Extract of P. ramosissima

**Figure 5:**
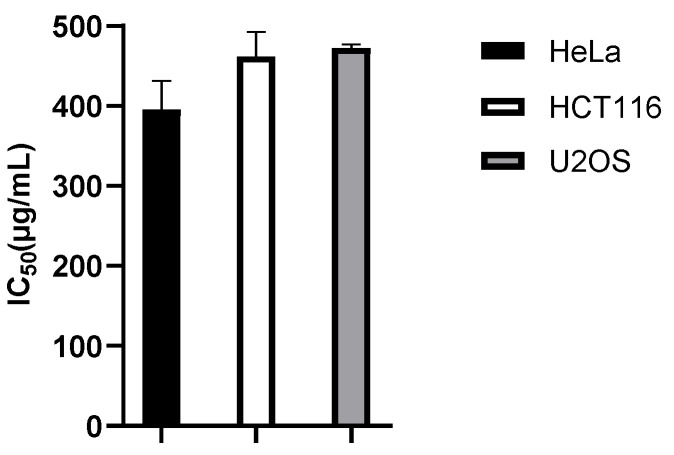
Antiproliferative activity of methanol extract of *P. ramosissima* on HeLa, HCT116, and U2OS cancer cell lines expressed as mean IC_50_ values after three independent experiments ± SD (standard deviation). Statistical analysis was performed using a one-way ANOVA test followed by Tukey’s multiple comparison test. No statistically significant difference was found.

The antiproliferative effect of volatile compounds is described in a very limited number of scientific papers. The promising results of previous studies dealing with this activity of aromatic plants [30] and the fact that the antiproliferative activity of *P. ramosissima* has not been previously investigated prompted us to examine the effect of hydrosol and methanol extract of this plant on cervical cancer cell line (HeLa), human colon cancer cell line (HCT116) and human osteosarcoma cell line (U2OS). The results showed that the hydrosol had no effect on the survival of the tested cells, although myristicin has shown significant biological activity, including antiproliferative properties, in the past [14]. Considering that hydrosols are generally less concentrated, it is possible that the concentration of bioactive compounds in the water extract is not sufficient to inhibit the growth of the tested cells. Treatment with serial dilutions of the methanol extract for 48 h moderately inhibited growth of all tested cancer cell lines with IC_50_ values for HeLa 395.73 µg/mL, HCT116 462.054 µg/mL, and U2OS 472.519 µg/mL (Figure 5). Chemical analysis of the methanol extract revealed several compounds, three flavonoid glycosides, one anthocyanidin glycoside (peonidin-3-rutinoside or peonidin-3-rhamnoside-5-glucoside), and the most abundant lipid compound, sphingolipid hexadecasphinganin (Table 3). Sphingolipids isolated from *Cissus incisa* leaves showed excellent cytotoxic activity on six human cancer cells: PC3, Hep3B, HepG2, MCF7, A549, and HeLa [30]. Flavonoids are known to have anticancer activity in several ways: they interrupt the cell cycle, activate enzymes that scavenge reactive oxygen species, stimulate apoptosis and autophagy, and inhibit cancer cell proliferation [31]. The compound identified in the methanol extract of *P. ramossisima*, peonidin-3-rutinoside or peonidin-3-rhamnoside-5-glucoside, was previously shown by Chen et al. (2005) to be able to inhibit the growth of HS578T cancer cells and stimulating apoptosis. It also stopped the growth of Lewis lung carcinoma cells in vivo [32].

Considering only a part of the literature results dealing with the antiproliferative effect of Apiaceae and other aromatic plant species, it is noted that methanol and other solvents have been used to extract various bioactive compounds from the plants. Zengin et al. [20] studied the cytotoxic effects of methanol extract of seven Apiaceae species on HepG2, hepatocellular carcinoma cells, and a healthy cell line, S17. Among plants tested, only *C. macrospermum* showed higher toxicity to HepG2 cells than to healthy S17 cells. Bogucka-Kocka et al. [31] demonstrated that etheric extracts from the fruits of *Laserpitium krapffii* Crantz showed moderate cytotoxic activity against human acute promyelocytic leukemia cell lines (HL-60) and acute lymphoblastic leukemia cell lines (CEM/C1 and CCRF/CEM) in contrast to methanol extracts. The antiproliferative activity of hexane, ethyl acetate, and methanol extracts of *Seseli petraeum* M. Bieb. was tested on A549 human lung cancer cells. Although all prepared extracts showed significant antiproliferative activity, the hexane extract had the greatest ability to inhibit the growth of treated cells. The authors indicated that the mechanism of action of the extract was the induction of cell cycle arrest in the G0/G1 phase. The hexane extracts significantly induced apoptosis and DNA damage in A549 cells [32].

The results presented in this study provide new valuable insights into the various bioactivities of this plant and complement the biological activities of Apiaceae plants in general.

### 2.5. Antiphytoviral Activity

Recently, we have been confronted with the global problem of the excessive use of pesticides and other, mainly synthetic means for plant protection against pathogens. The resulting environmental and human health consequences prompt us to focus on natural resources from which we can learn ways to combat various diseases. Even though natural means of protection against plant pests can hardly compete with the effect of synthetic agents, mainly for economic reasons, we must not slacken our efforts to determine the most effective methods of plant protection that do not have harmful effects on the environment. In several of our previous articles, we have confirmed our hypothesis of the antiphytoviral activity of plant constituents, especially essential oils. More recently, in addition to essential oils, we have focused on hydrosols as they are harmless, non-toxic aqueous solutions of volatiles that are by-products of essential oil distillation that can be easily and safely applied to plants. Knowing that plants of the Apiaceae family are widely used for food, flavour, fragrance, and medicinal purposes and that the rediscovery of this family may give rise to a new generation of botanical chemicals for industrial applications [33], we decided to investigate the antiphytoviral potential of *P. ramosissima*, especially since the plant is a rich source of myristicin, which is known for its insecticidal activity [34]. The viral disease caused by TMV has serious impact on vegetable crops, reducing yield and affecting quality, making efficient, environmentally friendly antiviral agents of natural origin a challenge for TMV eradication and/or prevention of TMV infestation. Previous findings on the antiviral efficacy of aromatic plants [8] and the composition of *P. ramosissima* volatiles presented in this paper (Table 1, Table 2 and Table 3) have prompted us to conduct antiviral studies with essential oil and hydrosol of this plant species to increase our knowledge of the biological activities of the Apiaceae family and volatiles in general.

**Figure 6:**
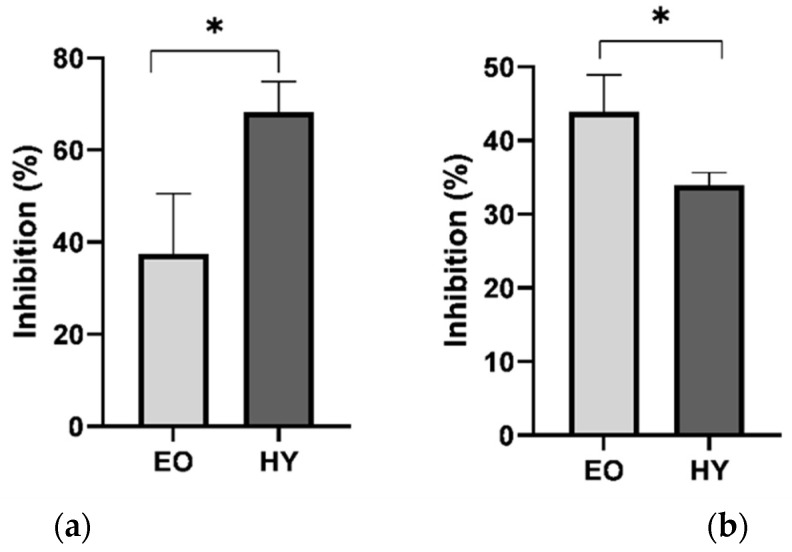
Antiphytoviral activity of *P. ramosissima* against tobacco mosaic virus infection on local host plants. (**a**) Pretreatment of plants with essential oil (EO) or hydrosol (HY). (**b**) Simultaneous inoculation of EO or HY with virus. * statistically significant differences between essential oil and hydrosol treatment data (*p* ˂ 0.05).

The results of the efficacy of EO and HY against TMV infection in local host plants are shown in Figure 6. Simultaneous inoculation of the virus with the tested extracts was effective for both EO and HY, although EO showed stronger antiviral activity with an inhibition rate of 43.9%. (Figure 6b). Both EO and HY showed a statistically significant reduction in the number of local lesions in the treated leaf halves (Table 4b). In a similar experiment where *Micromeria croatica* essential oil was incubated with viral inocula for 0, 1, 2, and 3 h before inoculation and then tested against satellite-associated cucumber mosaic virus, the antiviral activity did not exceed 40% [8]. The efficacy of HY has generally never been tested by simultaneous inoculation with viral inocula, and thus the antiviral activity of HY, which reached 34.0%, provides new insight into the possibility of its effect on viral infections. We also investigated the effects of the pretreatment of EO and HY on the defence response of local host plants to TMV infection. Although plants treated with both extracts before viral infection reduced the number of local lesions compared with control plants, only the reduction achieved by hydrosol treatment was statistically significant (Table 4a), reaching a promising inhibition rate of 68.2% (Figure 6a). This activity of EO and HY could be explained by the fact that essential oils, when inoculated simultaneously with the virus, are likely to cause direct inactivation of the viral particles, as opposed to stimulation of the host’s defence response, as assumed for the activity of HY during pretreatment. It has been documented that the main mechanism of antiviral effects of many essential oils against respiratory viruses may be related to capsid or membrane disintegration, and that essential oils and their components may also inhibit the late stages of the viral life cycle by interfering with the redox signalling pathway [35]. Comparison of previous experiments dealing with the antiphytoviral activity of essential oils and hydrosols of different plant species [8] with the antiviral activity of *P. ramosissima* extracts shows that EO and HY of this plant are a valuable source of compounds that can be further explored for the development of natural antiviral agents. In this context, we particularly highlight the activity of hydrosol, as our results have shown that volatile substances in the form of environmentally friendly water solutions are a promising new source of compounds that can be used to prevent the attack of viral pathogens.

## 3. Materials and Methods

### 3.1. Herbal Material

Aboveground plant parts were harvested before the flowering stage in a rocky habitat, at the locality Klis, Croatia (43°33′41.7′′ N, 16°31′39.1′′ E). The identity of the plant material was confirmed by PhD Juraj Kamenjarin based on the literature [2,36]. Voucher specimens of the plant material were deposited at the Faculty of Science, Department of Biology, University of Split, Split, Croatia. The plant material (collected in May 2018 and May 2019) was air-dried in a single layer for two weeks and mixture was packed in paper bags and stored in a dry place protected from light until hydrodistillation. The randomized mixture of fresh samples (collected in May 2020) was used the day after harvest for HS-SPME analysis of volatiles.

### 3.2. Hydrodistillation

An amount of 50.004 g of the dried plant material was mixed with 500 mL of water in the flask of the Clevenger apparatus; then, 35 mL of water and 2 mL of pentane (VWR Chemicals, Radnor, PA, USA) were added to the inner tube of the Clevenger apparatus. After hydrodistillation for 3 h, the fractions of hydrophobic (essential oil) and hydrophilic volatile compounds (extracted in pentane (used as the solvent trap) and water fractions, respectively) were removed from the apparatus separately and stored at −20 °C and +4 °C, respectively.

### 3.3. Methanol Extract

An amount of 1.002 g of dried plant material was freeze-dried and homogenized in 80% methanol-water followed by extraction in an ultrasonic bath at room temperature for 20 min. Then, 15 mL of 80% methanol–water was added and the mixture was centrifuged at 5000× *g* for 5 min. The supernatant was transferred to a new Eppendorf tube, centrifuged again at 7000× *g* for 20 min, transferred to a new tube, evaporated to dry in a rotary evaporator, dissolved in 10% DMSO and stored at −20 °C until use.

### 3.4. Headspace Solid-Phase Microextraction (HS-SPME)

Two SPME fibres (Agilent Technologies, Palo Alto, Santa Clara, CA, USA) used for HS-SPME covered with either PDMS/DVB (polydimethylsiloxane/divinylbenzene) or DVB/CAR/PDMS (divinylbenzene/carboxene/polydimethylsiloxane) were set on the PAL Auto Sampler System (PAL RSI 85, CTC Analytics AG, Schlieren, Switzerland). Before the analysis, both fibres were conditioned according to the manufacturer’s instructions. Glass vials with a volume of 20 mL were filled with 1 g of the plant samples or hydrosol and the vials were sealed with a stainless steel cap with polytetrafluorethylene (PTFE)/silicon septa. Equilibration of the sample was carried out at 60 °C for 15 min. The extraction was continued for 45 min, followed by 6 min of thermal desorption at an injection temperature of 250 °C directly into the GC column.

### 3.5. Gas Chromatography-Mass Spectrometry Analysis (GC-MS)

VOCs isolated from *P. ramosissima* were analyzed using a gas chromatograph (type 8890 Agilent Technologies, Palo Alto, Santa Clara, CA, USA) and a tandem mass spectrometer detector (type 5977E MSD, Agilent Technologies). The HP-5MS capillary column (30 m × 0.25 mm, 0.25 µm film thickness, Agilent Technologies, Palo Alto, Santa Clara, CA, USA) was used to separate VOCs. The conditions for GC-MS analysis and the procedure for compound identification were described in detail previously by Radman et al. [37]. Results for all samples were measured in three independent analyses and expressed as the average area percentage (%) of each compound.

### 3.6. Ultra High-Performance Liquid Chromatography—High-Resolution Mass Spectrometry (UHPLC-ESI-HRMS) of Methanol Extract

The UHPLC-ESI-HRMS analyses were performed on the UHPLC ExionLC AD system (AB Sciex, Concord, ON, Canada) equipped with the ExionLC modules: Controller, AD Pump, AD Degasser, solvent delivery system, AD Autosampler, AD Column oven tandem quadrupole time-of-flight (Q-TOF) mass spectrometer TripleTOF 6600+ (AB Sciex, Concord, Canada) containing Duospray ion source. The chromatographic separations of the compounds were carried through the analytical column Acquity UPLC BEH Phenyl-Hexyl (Waters, Milford, MA, USA; dimensions 2.1 mm × 100 mm and particle size 1.7 µm). Both mobile phases, water (A) and acetonitrile (B), contained 0.1% formic acid and were pumped with a continuous flow rate of 0.4 mL/min. The oven temperature was constantly set at 30 °C. The elution started isocratic with 2% of B (0.6 min) followed by the gradient program: 0.6–18.5 min (B linear gradient to 100%), 18.5–25 min (100% B). An amount of 4 µL of the sample was injected [38].

Electrospray ionisation was set in positive mode (ESI+) with the collision-induced dissociation (CID) in information-dependent acquisition (IDA) mode for MS/MS mass spectra recording. A detailed description of the parameters could be seen in Radman et al. [38].

Mass spectrometer data were processed using ACD/Spectrus Processor 2021.1.0. (ACD/Labs, Toronto, Canada) and the identification of the compounds was performed based on their mass spectra and the elemental compositions which were combined with the results of the search in the ChEBI, Lipid maps and MassBank database.

### 3.7. Antiproliferative Analysis

The antiproliferative analysis of methanol extract of *P. ramosissima* on three cancer cell lines, cervical cancer cell line (HeLa), human colon cancer cell line (HCT116), and human osteosarcoma cell line (U2OS) was performed according to the protocol described in our previously published papers [39] using the MTS-based CellTiter 96^®^ Aqueous Assay (Promega, Madison, WI, USA). Cells were donated by prof. Janoš Terzić from the School of Medicine, University of Split. Cells were grown in a CO_2_ incubator at 37 °C and 5% CO_2_ until they reached 80% confluency. They were further counted using the automatic handheld cell counter (Merck, Darmstadt, Germany), 5000 cells per well were seeded in 96-well plates and treated with serially diluted methanolic extract of *P. ramosissima*. The cells were cultured for an additional 48 h, after which 20 µL of MTS tetrazolium reagent (Promega, Madison, WI, USA) was added to each well and left for 3 h in the incubator at 37 °C and 5% CO_2_. The absorbance was measured at 490 nm using a 96-well plate reader (Bio-Tek, EL808, Winooski, VT, USA). Measurements were performed in four replicates for each concentration and IC_50_ values were calculated from three independent experiments.

### 3.8. Antiphytoviral Activity Assay

Leaves of *Nicotiana tabacum* L. cv. Samsun systemically infected with TMV were used to prepare the virus inocula. The species *Datura stramonium* L. was used as a local host for TMV since it develops clearly visible lesions 3–4 days after inoculation. The leaves of *D. stramonium* were dusted with silicon carbide (Sigma-Aldrich, St. Louis, MO, USA) to produce microscopic wounds during mechanical inoculation through which viruses could enter plant cells. The prepared inocula were diluted with inoculation buffer to obtain 15–50 lesions per inoculated leaf. The experiments were conducted when the plants reached the 5–6 leaf stage. Care was taken to ensure that the experimental plants were as uniform in size as possible. For pretreatment, essential oil (500 ppm) and hydrosol (undiluted) were applied as a spray solution to the leaves of *D. stramonium* plants for two consecutive days. Subsequently, the leaves were inoculated with a freshly prepared inoculum. The antiphytoviral activity of the tested extracts was evaluated by the percentage inhibition of the number of local lesions on the leaves of the treated and control plants. For simultaneous inoculation, half of the leaves of local host plants were rubbed with virus inocula (1 mL) to which essential oil (1 µL) or hydrosol (100 µL) was added. The opposite (control) leaf halves were inoculated with the same concentration of viral inocula. The percentage of inhibition was calculated by comparing the number of lesions on the control and treated leaf halves [8].

### 3.9. Statistical Analysis

Statistical analysis was performed in GraphPad Prism Version 9. All data are expressed as mean ± SD (*n* = 3). Statistical significance was assessed by *t*-test (antiphytoviral activity) and one-way ANOVA followed by Turkey’s multiple comparison test (antiproliferative activity). Differences were considered significant at * *p* < 0.05.

## 4. Conclusions

We have presented a comprehensive study of the volatile and non-volatile organic compounds of the Mediterranean endemic species *Portenschlagiella ramosissima* (Port.) Tutin. The essential oil and hydrosol as well as the fresh and dry plant material are rich in myristicin, which was detected as the predominant compound after hydrodistillation in EO and HY and in the fresh and dry plant material after HS-SPME extraction. This analysis, using two fibres for the first time, provides a complete insight into the volatile composition of this plant species. Among the non-volatile compounds of the methanol extract, three flavonoid glycosides, one anthocyanidin glycoside and lipid derivatives were detected. The methanol extract moderately inhibited the growth of cancer cell lines: HeLa, HCT116, and U2OS. The antiphytoviral activity of both the essential oil and hydrosol suggests that *P. ramosissima* volatiles are a promising new source of compounds that can be used to prevent attack by viral pathogens. This endemic and poorly studied plant is thus an available source of new compounds with a range of beneficial effects, the spectrum and mechanism of action of which could be complemented by new knowledge in the future.

## Figures and Tables

**Table 1:** Volatile organic compounds of air-dried *P. ramosissima* isolated by hydrodistillation and analyzed by gas chromatography-mass spectrometry.

No.	Compound	RI	Average Area% ± SD	
			EO	HY
1	Furfural	<900	-	0.40 ± 0.08
2	(*E*)-Hex-2-enal	<900	0.03 ± 0.03	0.11 ± 0.03
3	Ethylbenzene	<900	-	0.56 ± 0.00
4	p-Xylene	<900	-	2.24 ± 0.04
5	Nonane	901	0.48 ± 0.10	-
6	(*Z*)-Non-2-ene	917	0.02 ± 0.00	-
7	α-Thujene	934	0.12 ± 0.03	-
8	α-Pinene	942	0.06 ± 0.01	-
9	Benzaldehyde	966	0.01 ± 0.00	0.18 ± 0.02
10	Sabinene	982	10.23 ± 0.33	-
11	β-Pinene	985	0.26 ± 0.06	-
12	β-Myrcene	994	0.68 ± 0.13	-
13	Decane	1001	0.02 ± 0.00	-
14	α-Phellanderene	1009	0.01 ± 0.00	-
15	δ-3-Carene	1016	0.01 ± 0.00	-
16	α-Terpinene	1023	0.34 ± 0.08	-
17	*p*-Cymene	1031	0.85 ± 0.18	-
18	β-Phellandrene	1036	0.09 ± 0.02	-
19	Eucalyptole	1038	0.01 ± 0.00	
20	Benzyl alcohol	1040	-	0.25 ± 0.01
21	(*E*)-β-Ocimene	1045	7.95 ± 0.12	-
22	Phenylacetaldehyde	1051	0.03 ± 0.01	0.86 ± 0.01
23	(*Z*)-β-Ocimene	1054	0.29 ± 0.06	-
24	γ-Terpinene	1066	2.80 ± 0.38	-
25	2-Methyldecane	1068	0.01 ± 0.00	-
26	(*E*)-4-Thujanol	1073	0.17 ± 0.04	2.09 ± 0.19
27	Linalool oxide	1078	-	0.10 ± 0.01
28	α-Terpinolene	1092	0.25 ± 0.05	0.18 ± 0.03
29	Linalool	1101	-	3.60 ± 0.23
30	Undecane	1102	1.72 ± 0.31	-
31	2,6-Dimethylcyclohexan-1-ol	1113	-	0.27 ± 0.05
32	2-Phenylethanol	1118	-	0.41 ± 0.04
33	(*Z*)-*p*-Menthen-2-en-1-ol	1127	0.07 ± 0.02	0.97 ± 0.12
34	(*Z*)-Alloocimene	1134	0.11 ± 0.03	-
35	Terpinen-1-ol	1146	0.03 ± 0.01	0.68 ± 0.07
36	*p*-Methoxystyrene	1158	0.01 ± 0.00	-
37	Sabine ketone	1164	-	0.10 ± 0.02
38	Terpinen-4-ol	1183	0.99 ± 0.20	11.36 ± 0.59
39	*p*-Cymen-8-ol	1189	-	0.27 ± 0.00
40	α-Terpineol	1194	0.04 ± 0.01	0.77 ± 0.03
41	(*Z*)-Dec-4-enal	1197	0.06 ± 0.02	-
42	(*Z*)-Piperitol	1198	-	0.14 ± 0.01
43	Dodecane	1202	0.04 ± 0.01	-
44	(3*Z*,5*Z*)-2,6-Dimethylocta-3,5,7-trien-2-ol	1204	0.02 ± 0.01	0.12 ± 0.02
45	Decanal	1209	0.06 ± 0.01	-
46	(3*E*,5*E*)-2,6-Dimethylocta-3,5,7-trien-2-ol	1213	0.04 ± 0.01	-
47	(*E*)-Piperitol	1213	-	0.35 ± 0.04
48	Benzothiazole	1228	-	0.35 ± 0.03
49	Thymol methyl ether	1241	2.32 ± 0.72	-
50	Carvacrol methyl ether	1250	0.03 ± 0.01	-
51	Dec-4-en-1-ol	1264	0.06 ± 0.03	-
52	2-Phenylbut-2-enal	1277	-	0.11 ± 0.01
53	(*Z*)-Tridec-3-ene	1295	0.01 ± 0.00	-
54	Indole	1296	-	0.09 ± 0.01
55	Tridecane	1302	0.01 ± 0.01	-
56	Thymol	1306	0.01 ± 0.00	-
57	Undecanal	1310	0.01 ± 0.01	-
58	*p*-Vinylguaiacol (4-Ethenyl-2-methoxyphenol)	1318	0.01 ± 0.00	0.39 ± 0.10
59	Cyclosativene	1372	0.02 ± 0.01	-
60	α-Copaene	1380	0.03 ± 0.01	-
61	β-Elemene	1395	0.02 ± 0.01	-
62	Methyleugenol	1409	0.72 ± 0.09	0.45 ± 0.03
63	Dodecanal	1413	0.27 ± 0.05	-
64	β-Funebrene	1416	0.06 ± 0.01	-
65	Caryophyllene	1424	2.16 ± 0.36	-
66	α-Gurjunene	1429	0.03 ± 0.01	-
67	β-Gurjunene	1434	0.01 ± 0.00	-
68	α-Humulene	1458	0.22 ± 0.05	-
69	β-Farnesene	1462	0.04 ± 0.01	-
70	γ-Curcumene	1471	0.01 ± 0.00	-
71	γ-Muurolene	1478	0.03 ± 0.00	-
72	Germacrene D	1485	0.35 ± 0.09	0.06 ± 0.01
73	Germacrene C	1499	0.05 ± 0.01	-
74	α-Muurolene	1503	0.06 ± 0.02	-
75	Myristicin	1538	63.92 ± 3.80	66.67 ± 1.45
76	Elemicin (1,2,3-Trimethoxy-5-prop-2-enylbenzene)	1566	0.82 ± 0.03	5.13 ± 0.04
77	Caryophyllene oxide	1589	-	0.06 ± 0.01
78	(*E*)-Isomyristicin	1621	0.04 ± 0.00	-
79	Cubenol	1648	0.05 ± 0.01	-
80	(*E*)-Isoelemicin	1658	0.01 ± 0.00	0.15 ± 0.02
81	α-Cadinol	1661	0.07 ± 0.01	-
82	Benzyl benzoate	1768	0.06 ± 0.01	-
83	Neophytadiene	1844	0.01 ± 0.00	-
84	Phytol	2120	0.13 ± 0.09	-

EO—essential oil of *P. ramosissima*; HY—hydrosol of *P. ramosissima*. SD is the standard deviation of a triplicate sample; RI—retention index.

**Table 2:** Volatile organic compounds isolated by headspace solid-phase microextraction and analyzed by gas chromatography–mass spectrometry from *P. ramosissima* samples.

No.	Compound	RI	Average Area% ± SD					
			HS-Fr (f1)	HS-Dr (f1)	HS-HY (f1)	HS-Fr (f2)	HS-Dr (f2)	HS-HY (f2)
1	Pentanal	<900	-	-	0.04 ± 0.02	-	-	0.1 ± 0.00
2	Pyridine	<900	-	-	0.04 ± 0.01	-	-	0.05 ± 0.01
3	Furfural	<900	-	-	0.10 ± 0.01	-	-	0.16 ± 0.02
4	(*E*)-Hex-2-enal	<900	-	-	-	0.03 ± 0.01	-	-
5	Hexan-1-ol	<900	-	-	0.02 ± 0.00	-	-	0.02 ± 0.01
6	Nonane	904	0.09 ± 0.00	0.15 ± 0.02	-	0.14 ± 0.01	0.07 ± 0.01	-
7	(*Z*)-Non-2-ene	920	-	0.01 ± 0.00	-	0.01 ± 0.00	-	-
8	α-Thujene	937	0.24 ± 0.02	0.32 ± 0.02	-	0.13 ± 0.00	0.13 ± 0.03	-
9	α-Pinene	945	0.02 ± 0.00	0.03 ± 0.00	0.03 ± 0.00	0.03 ± 0.00	0.02 ± 0.00	0.02 ± 0.01
10	Benzaldehyde	970	-	0.01 ± 0.00	0.10 ± 0.01	-	0.01 ± 0.00	0.10 ± 0.03
11	Sabinene	982	4.07 ± 0.29	5.32 ± 0.27	0.04 ± 0.01	7.70 ± 0.56	3.99 ± 0.33	0.03 ± 0.01
12	β-Pinene	986	0.27 ± 0.00	0.37 ± 0.02	-	0.27 ± 0.01	0.19 ± 0.05	-
13	β-Myrcene	995	0.49 ± 0.04	0.46 ± 0.02	-	0.50 ± 0.01	0.24 ± 0.07	-
14	Decane	1003	-	0.04 ± 0.00	-	-	0.01 ± 0.00	-
15	2,6-Dimethylcyclohexan-1-ol	1009	-	-	0.03 ± 0.01	-	-	-
16	[(*Z*)-Hex-3-enyl] acetate	1011	0.44 ± 0.07	-	-	-	-	-
17	α-Phellanderene	1011	-	0.02 ± 0.00	0.08 ± 0.01	0.01 ± 0.00	-	0.07 ± 0.03
18	Hexyl acetate	1018	0.07 ± 0.01	-	-	-	-	-
19	α-Terpinene	1023	0.22 ± 0.02	0.11 ± 0.00	0.10 ± 0.00	0.09 ± 0.01	0.04 ± 0.01	0.09 ± 0.03
20	*p*-Cymene	1032	0.54 ± 0.01	1.21 ± 0.03	-	0.87 ± 0.03	0.68 ± 0.32	-
21	β-Phellandrene	1037	0.12 ± 0.01	0.09 ± 0.00	0.12 ± 0.00	0.06 ± 0.00	0.04 ± 0.01	0.07 ± 0.02
22	Benzyl alcohol	1041	-	0.02 ± 0.00	0.04 ± 0.00	-	0.10 ± 0.08	0.08 ± 0.03
23	(*E*)-β-Ocimene	1045	8.76 ± 0.95	8.73 ± 0.23	-	9.05 ± 0.11	5.10 ± 1.78	-
24	Phenylacetaldehyde	1051	0.03 ± 0.01	-	0.12 ± 0.03	-	-	0.16 ± 0.01
25	(*Z*)-β-Ocimene	1055	0.31 ± 0.03	0.20 ± 0.01	-	0.25 ± 0.00	0.11 ± 0.03	-
26	γ-Terpinene	1066	2.05 ± 0.24	2.00 ± 0.06	0.09 ± 0.00	1.94 ± 0.00	1.10 ± 0.45	0.05 ± 0.02
27	2-Methyldecane	1069	-	0.03 ± 0.00	-	-	0.02 ± 0.00	-
28	(*E*)-4-Thujanol	1074	0.04 ± 0.01	0.18 ± 0.00	0.68 ± 0.06	0.08 ± 0.00	0.12 ± 0.09	0.68 ± 0.16
29	α-Terpinolene	1093	0.09 ± 0.01	0.09 ± 0.00	0.03 ± 0.00	0.07 ± 0.00	0.05 ± 0.02	-
30	Nonanal	1097	0.01 ± 0.00	0.03 ± 0.00	-	-	0.01 ± 0.00	-
31	Methyl benzoate	1099	0.01 ± 0.00	-	-	-	-	-
32	Linalool	1102	-	-	0.80 ± 0.08	-	-	0.77 ± 0.18
33	Undecane	1103	2.10 ± 0.49	4.15 ± 0.14	-	1.06 ± 0.05	2.13 ± 0.68	-
34	2-Phenylethanol	1119	-	-	0.05 ± 0.00	-	-	-
35	(3*E*,5*Z*)-2,6-Dimethylocta-1,3,5,7-tetraene	1128	0.04 ± 0.00	0.03 ± 0.00	-	0.04 ± 0.00	0.02 ± 0.01	-
36	(*Z*)-*p*-Menthen-2-en-1-ol	1128	-	-	0.32 ± 0.03	-	-	0.33 ± 0.08
37	(*Z*)-Alloocimene	1134	0.24 ± 0.02	0.24 ± 0.01	0.03 ± 0.00	0.19 ± 0.01	0.11 ± 0.05	-
38	Terpinen-1-ol	1146	-	-	0.21 ± 0.02	-	-	0.20 ± 0.06
39	3-Methylundecane	1175	0.04 ± 0.01	0.06 ± 0.00	-	-	0.04 ± 0.00	-
40	Terpinen-4-ol	1183	0.06 ± 0.02	0.09 ± 0.00	5.31 ± 0.54	0.16 ± 0.05	0.06 ± 0.04	4.72 ± 0.89
41	*p*-Cymen-8-ol	1190	-	-	0.05 ± 0.00	-	-	0.07 ± 0.01
42	*α*-Terpineol	1195	-	-	0.25 ± 0.03	-	-	0.24 ± 0.06
43	(*Z*)-Dec-4-enal	1197	0.01 ± 0.00	-	-	0.05 ± 0.00	-	-
44	Salicylic acid	1198	-	0.02 ± 0.00	-	-	0.01 ± 0.00	-
45	(*Z*)-Piperitol	1199	-	-	0.09 ± 0.00	-	-	0.09 ± 0.00
46	Dodecane	1202	-	0.01 ± 0.00	-	-	0.01 ± 0.00	-
47	(3*Z*,5*Z*)-2,6-Dimethylocta-3,5,7-trien-2-ol	1205	-	0.04 ± 0.00	-	-	0.03 ± 0.01	-
48	Decanal	1209	0.02 ± 0.00	-	-	0.06 ± 0.01	-	-
49	(3*E*,5*E*)-2,6-Dimethylocta-3,5,7-trien-2-ol	1213	-	0.01 ± 0.00	-	-	0.02 ± 0.00	-
50	(*E*)-Piperitol	1213	-	-	0.10 ± 0.01	-	-	0.10 ± 0.01
51	Thymol methyl ether	1236	1.38 ± 0.05	2.14 ± 0.00	0.03 ± 0.00	1.71 ± 0.04	1.75 ± 0.53	0.03 ± 0.00
52	Carvacrol methyl ether	1250	0.02 ± 0.00	0.04 ± 0.00	-	0.02 ± 0.00	0.03 ± 0.01	-
53	Dec-4-en-1-ol	1263	-	0.04 ± 0.00	-	-	0.03 ± 0.00	-
54	2-Phenylbut-2-enal	1277	-	-	0.05 ± 0.00	-	-	0.04 ± 0.00
55	(*Z*)-Tridec-3-ene	1293	0.02 ± 0.00	0.03 ± 0.00	-	-	0.02 ± 0.01	-
56	Tridecane	1302	0.04 ± 0.01	0.05 ± 0.00	-	0.01 ± 0.00	0.03 ± 0.01	-
57	Thymol	1306	-	-	0.02 ± 0.00	-	-	-
58	*p*-Vinylguaiacol (4-Ethenyl-2-methoxyphenol)	1318	-	-	0.07 ± 0.00	-	-	0.06 ± 0.00
59	Cyclosativene	1372	0.01 ± 0.00	0.04 ± 0.00	-	-	0.03 ± 0.01	-
60	α-Copaene	1380	0.02 ± 0.01	0.06 ± 0.00	-	-	0.05 ± 0.00	-
61	β-Elemene	1392	-	0.01 ± 0.00	0.11 ± 0.01	-	0.01 ± 0.00	0.09 ± 0.00
62	Methyleugenol	1409	0.29 ± 0.01	0.26 ± 0.00	0.36 ± 0.00	0.22 ± 0.00	0.36 ± 0.06	0.29 ± 0.01
63	Dodecanal	1413	0.15 ± 0.03	0.02 ± 0.00	-	0.14 ± 0.01	0.11 ± 0.09	-
64	β-Funebrene	1416	0.16 ± 0.03	0.17 ± 0.00	-	0.06 ± 0.00	0.15 ± 0.03	-
65	β-Caryophyllene	1424	5.71 ± 1.03	4.60 ± 0.06	-	1.58 ± 0.12	4.53 ± 0.29	-
66	α-Gurjunene	1429	0.04 ± 0.01	0.03 ± 0.00	-	0.02 ± 0.00	0.03 ± 0.00	-
67	β-Gurjunene	1433	0.03 ± 0.01	0.03 ± 0.00	-	-	0.03 ± 0.00	-
68	α-Humulene	1458	0.41 ± 0.08	0.33 ± 0.00	-	0.12 ± 0.02	0.32 ± 0.03	-
69	β-Farnesene	1462	0.09 ± 0.03	0.10 ± 0.00	-	0.03 ± 0.00	0.10 ± 0.01	-
70	γ-Curcumene	1471	0.02 ± 0.00	0.02 ± 0.00	-	-	0.02 ± 0.00	-
71	γ-Muurolene	1478	0.04 ± 0.01	0.05 ± 0.00	-	0.01 ± 0.00	0.04 ± 0.01	-
72	α-Curcumene	1484	0.43 ± 0.12	0.16 ± 0.00	-	0.16 ± 0.00	0.27 ± 0.1	-
73	Germacrene D	1487	-	0.06 ± 0.00	-	-	0.06 ± 0.01	-
74	Germacrene C	1497	0.04 ± 0.01	0.03 ± 0.00	-	-	0.03 ± 0.00	-
75	α-Muurolene	1503	0.05 ± 0.01	0.08 ± 0.00	-	0.02 ± 0.00	0.07 ± 0.00	-
76	γ-Cadinene	1518	0.04 ± 0.01	0.04 ± 0.00	-	0.02 ± 0.00	0.04 ± 0.01	-
77	2,4-Di*tert*-butylphenol	1519	-	-	0.08 ± 0.04	-	-	0.08 ± 0.03
78	Myristicin	1533	69.99 ± 0.19	66.63 ± 0.77	88.03 ± 1.07	72.72 ± 0.54	76.57 ± 3.96	88.85 ± 1.99
79	Elemicin (1,2,3-trimethoxy-5-prop-2-enylbenzene)	1563	0.25 ± 0.00	0.20 ± 0.00	1.49 ± 0.09	0.19 ± 0.01	0.23 ± 0.01	1.44 ± 0.17
80	[(*Z*)-Hex-3-enyl] benzoate	1575	0.03 ± 0.00	-	0.03 ± 0.00	-	-	-
81	Caryophyllene oxide	1587	0.04 ± 0.00	0.32 ± 0.01	-	0.03 ± 0.00	0.28 ± 0.09	-
82	(*E*)-Isomyristicin	1620	0.06 ± 0.00	0.03 ± 0.00	0.15 ± 0.02	-	0.02 ± 0.01	0.12 ± 0.01
83	Cubenol	1647	-	-	0.04 ± 0.00	-	-	0.05 ± 0.01
84	α-Cadinol	1659	-	-	0.17 ± 0.01	-	-	0.18 ± 0.02
85	Benzyl benzoate	1768	-	-	0.03 ± 0.00	-	-	0.04 ± 0.01

Divinylbenzene/carboxene/polydimethylsiloxane fibre (f1); polydimethylsiloxane/divinylbenzene fibre (f2); HS-Fr—fresh *P. ramosissima*; HS-Dr—air-dried *P. ramosissima*; HS-HY—hydrosol of air-dried *P. ramosissima.*

**Table 3:** Major non-volatile compounds in methanol extract and their probable and possible structure and/or class identification by ultra-high-performance liquid chromatography–high-resolution mass spectrometry with electrospray ionisation.

	t_R_ (min)	Name	Monoisotopic Mass	[M + H]^+^	Mass Difference (ppm)	Structure	Area (Counts)
Flavonoids							
1	6.24	Flavonoid glycoside 1 (Isoquercitrin/Hyperoside/Quercetin/Spiraeoside) *	464.09548	465.10275	3.0	C_21_H_20_O_12_	600,867
2	6.71	Flavonoid glycoside 2 (Flavonol O-glycoside) *	448.10056	449.10784	3.5	C_21_H_20_O_11_	963,514
3	6.87	Flavonoid glycoside 3 (Flavone C-glycoside) *	462.11621	463.12349	5.0	C_22_H_22_O_11_	2,714,820
7	18.58	Anthocyanidin glycoside (peonidin 3-rutinoside or peonidin 3-rhamnoside 5-glucoside)	609.18195	610.18922	4.5	C_28_H_33_O_15_	2,846,789
Lipids							
4	7.68	Hexadecasphinganine	273.26678	274.27406	7.7	C_16_H_35_NO_2_	12,726,415
5	13.14	Octadecanoid	308.19876	309.20604	0.6	C_18_H_28_O_4_	1,505,124
6	16.02	Erucamide *	337.33446	338.34174	6.0	C_22_H_43_NO	3,888,486
8	19.05	Phosphoserine (PS (O-18:0/13:0) or PS (O-16:0/15:0)	707.51012	708.51740	5.2	C_37_H_74_NO_9_P	1,957,720

* additional MassBank verification.

**Table 4:** Number of local lesions on: (a) leaves of local host plants treated with essential oil and hydrosol before virus inoculation; (b) leaf halves simultaneously inoculated with virus and essential oil or hydrosol.

		LLN ± SD			LLN ± SD
(a)	C	52.17 ± 14.54	(b)	Ch	8.84 ± 0.92
	PT-EO	32.10 ± 9.53		SI-EO	4.98 ± 0.81 *
	PT-HY	16.67 ± 6.81 *		Ch	14.98 ± 1.89
				SI-HY	9.90 ± 1.40 *

LLN—local lesion number; (a) C—leaves of control plants; PT-EO—leaves of plants pretreated with essential oil; PT-HY leaves of plants pretreated with hydrosol; (b) Ch—control leaf halves; SI-EO—leaf halves simultaneously inoculated with virus and essential oil; SI-HY—leaf halves simultaneously inoculated with virus and hydrosol; SD—Standard deviation of triplicate analysis. Significant differences were determined by *t*-test; * statistically significant differences between control and treatment data (*p* ˂ 0.05).

## Data Availability

Data is contained within the article and in the Supplementary Materials.

## Supplementary Materials

The following supporting information can be downloaded at https://www.mdpi.com/article/10.3390/ph15121454/s1, Figure S1: UHPLC-ESI(+)—HRMS peak of compound 1, identified as flavonoid glycoside 1 (isoquercitrin/hyperoside/quercetin/spiraeoside): (a) full-scan MS; (b) MS^2^ spectrum; Figure S2: UHPLC-ESI(+)—HRMS peak of compound 2, identified as flavonoid glycoside 2 (flavonol O-glycoside): (a) full-scan MS; (b) MS^2^ spectrum; Figure S3: UHPLC-ESI(+)—HRMS peak of compound 3, identified as flavonoid glycoside 3 (flavone C-glycoside): (a) full-scan MS; (b) MS^2^ spectrum; Figure S4: UHPLC-ESI(+)—HRMS peak of compound 4, identified as Hexadecasphinganine—full-scan MS; Figure S5: UHPLC-ESI(+)—HRMS peak of compound 5, identified as octadecanoid: (a) full-scan MS; (b) MS^2^ spectrum; Figure S6: UHPLC-ESI(+)—HRMS peak of compound 6, identified as erucamide: (a) full-scan MS; (b) MS^2^ spectrum; Figure S7: UHPLC-ESI(+)—HRMS peak of compound 7, identified as anthocyanidin glycoside (peonidin 3-rutinoside or peonidin 3-rhamnoside 5-glucoside): (a) full-scan MS; (b) MS^2^ spectrum; Figure S8: UHPLC-ESI(+)—HRMS peak of compound 8, identified as phosphoserine (PS (O-18:0/13:0) or PS (O-16:0/15:0)): (a) full-scan MS; (b) MS^2^ spectrum.
