# Supplementary material for: A Plant Worthy of Further Study—Volatile and Non-Volatile Compounds of Portenschlagiella ramosissima (Port.) Tutin and Its Biological Activity
Source: Pharmaceuticals (Basel). 2022 Nov 23;15(12):1454. doi: 10.3390/ph15121454 (PMC9781946; doi:10.3390/ph15121454)
Supplement: Supplementary file 1 [file pharmaceuticals-15-01454-s001.zip › pharmaceuticals-1978654-supplementary.pdf]

Supplementary Materials

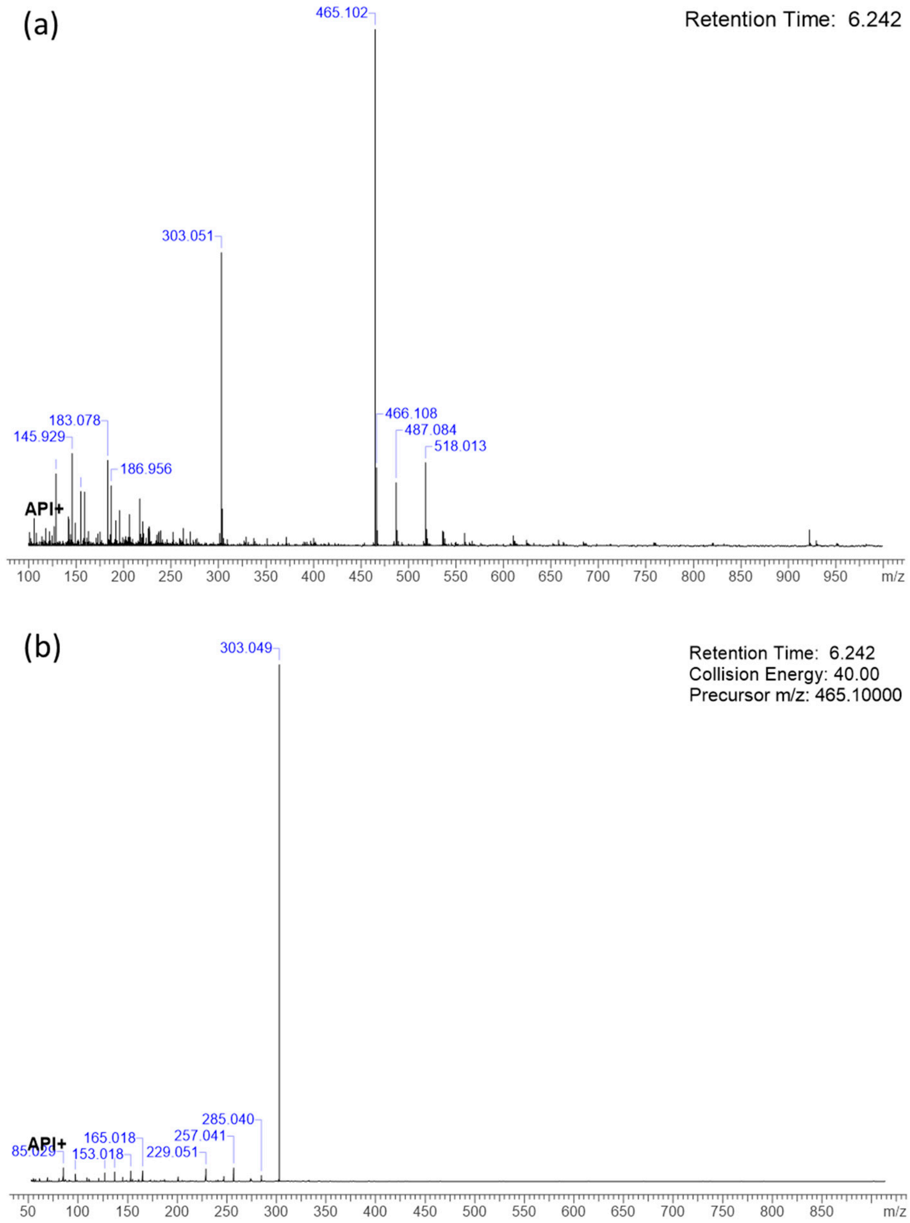

Figure S1. UHPLC-ESI(+)–HRMS peak of compound 1, identified as flavonoid glycoside 1 (isoquercitrin/hyperoside/quercetin/spiraeoside): (**a**) full-scan MS; (**b**) MS^2 spectrum.

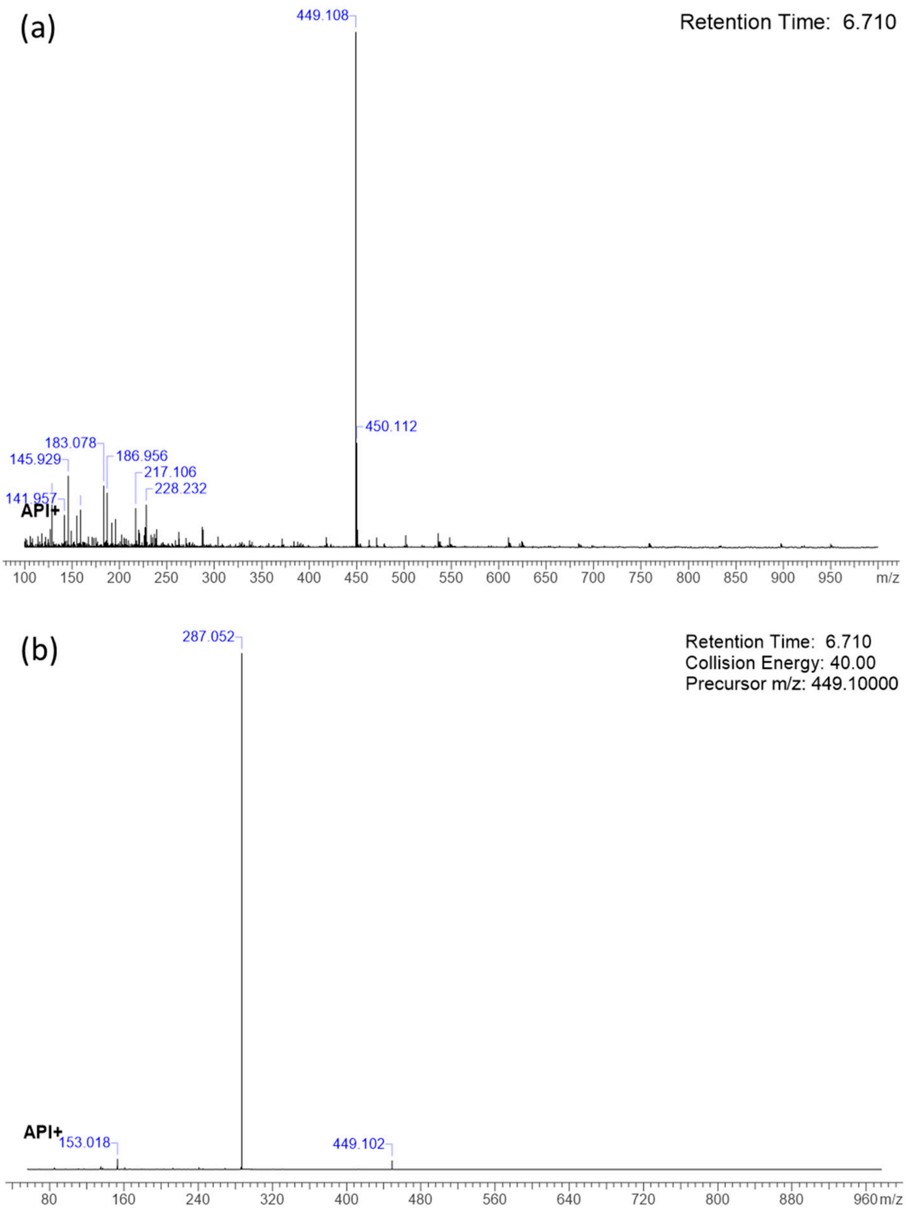

Figure S2. UHPLC-ESI(+)–HRMS peak of compound 2, identified as flavonoid glycoside 2 (flavonol O-glycoside): (**a**) full-scan MS; (**b**) MS^2 spectrum.

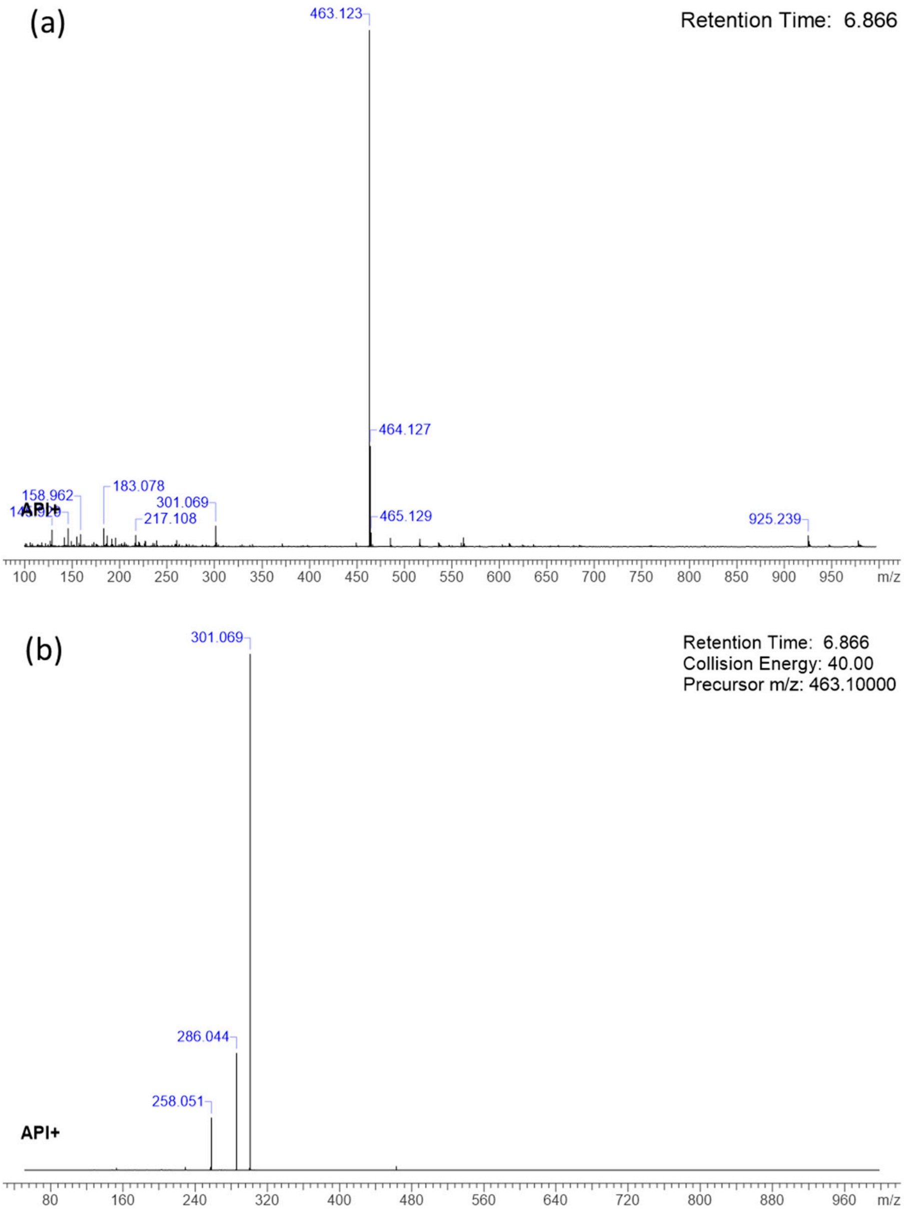

Figure S3. UHPLC-ESI(+)–HRMS peak of compound 3, identified as flavonoid glycoside 3 (flavone C-glycoside): (**a**) full-scan MS; (**b**) MS^2 spectrum.

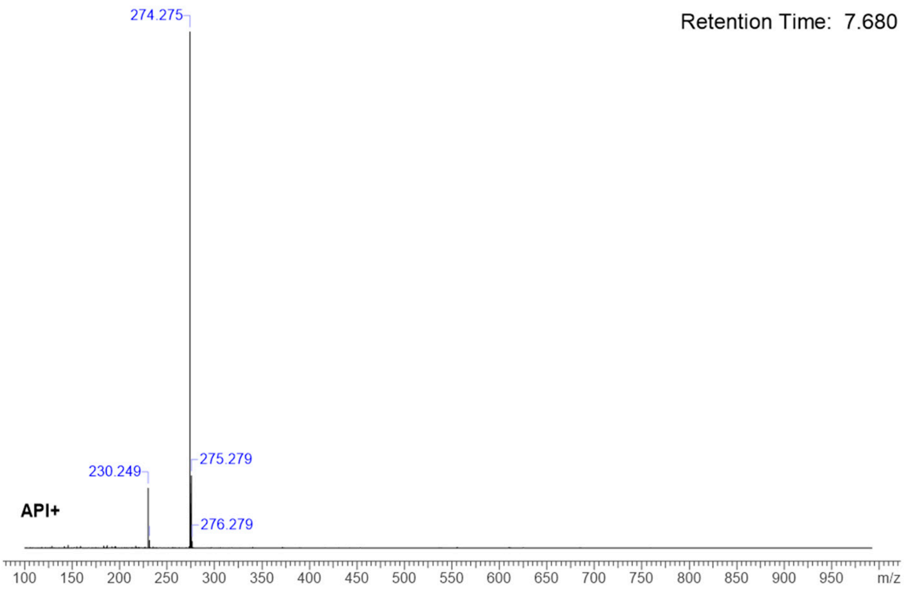

Figure S4. UHPLC-ESI(+)–HRMS peak of compound 4, identified as Hexadecasphinganine - full-scan MS.

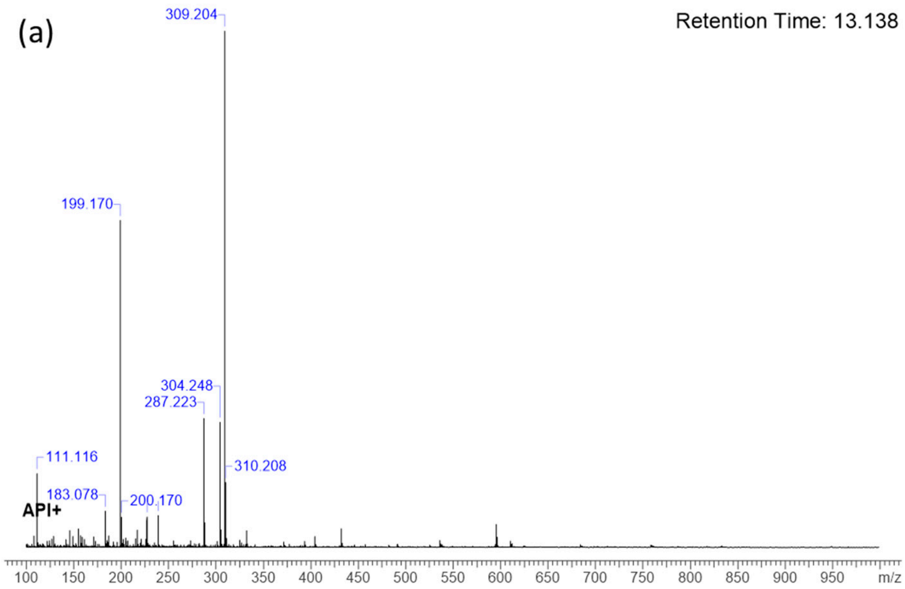

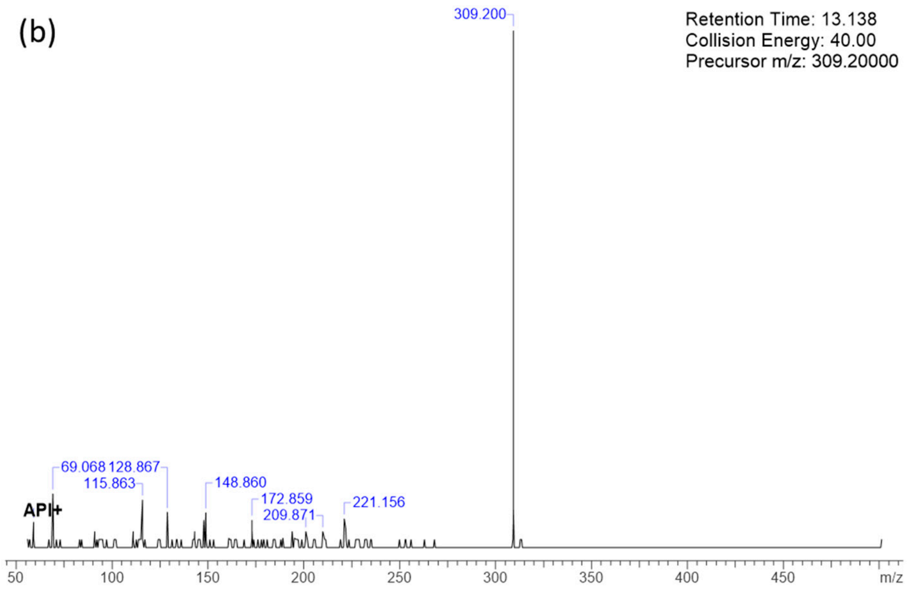

Figure S5. UHPLC-ESI(+)–HRMS peak of compound 5, identified as octadecanoid: (**a**) full-scan MS; (**b**) MS^2 spectrum.

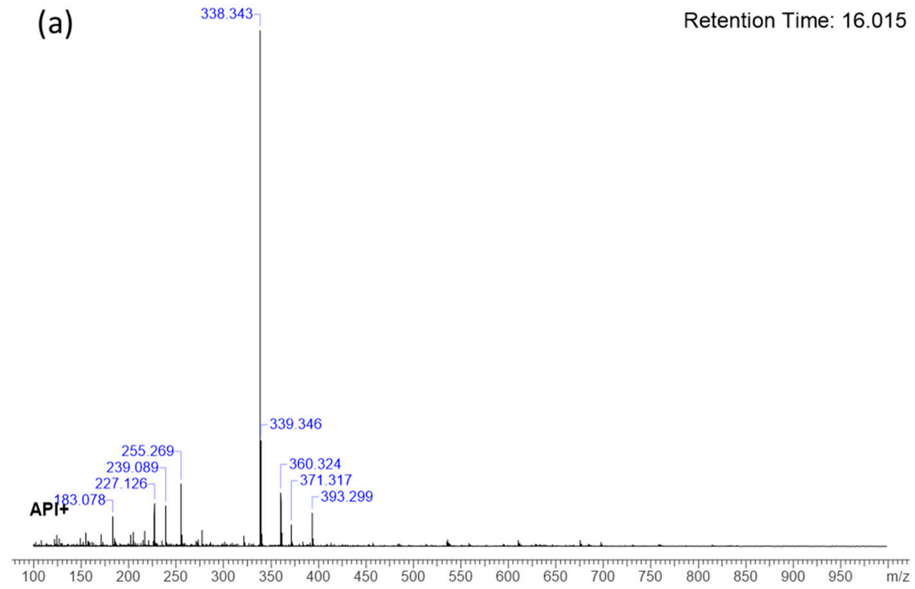

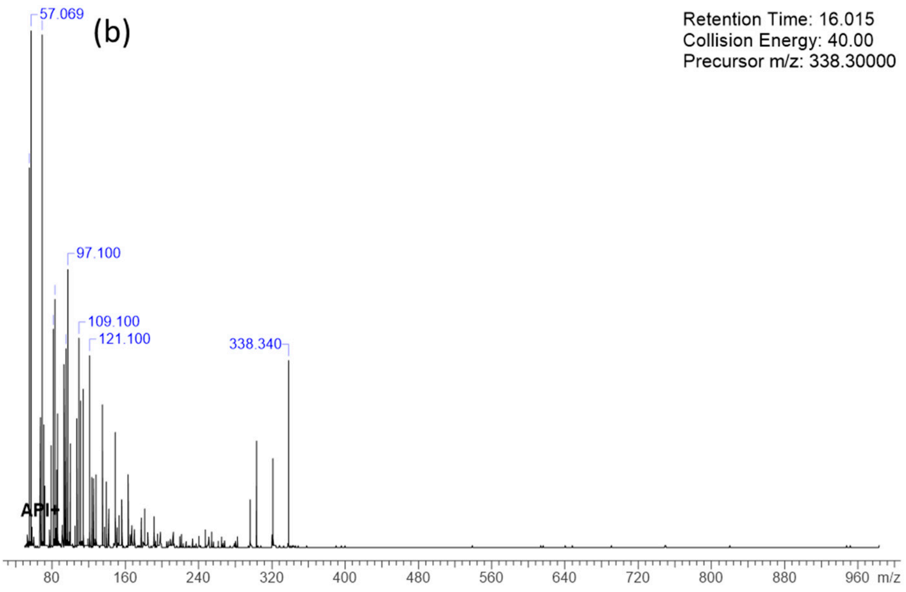

Figure S6. UHPLC-ESI(+)–HRMS peak of compound 6, identified as erucamide: (**a**) full-scan MS; (**b**) MS[2] spectrum.

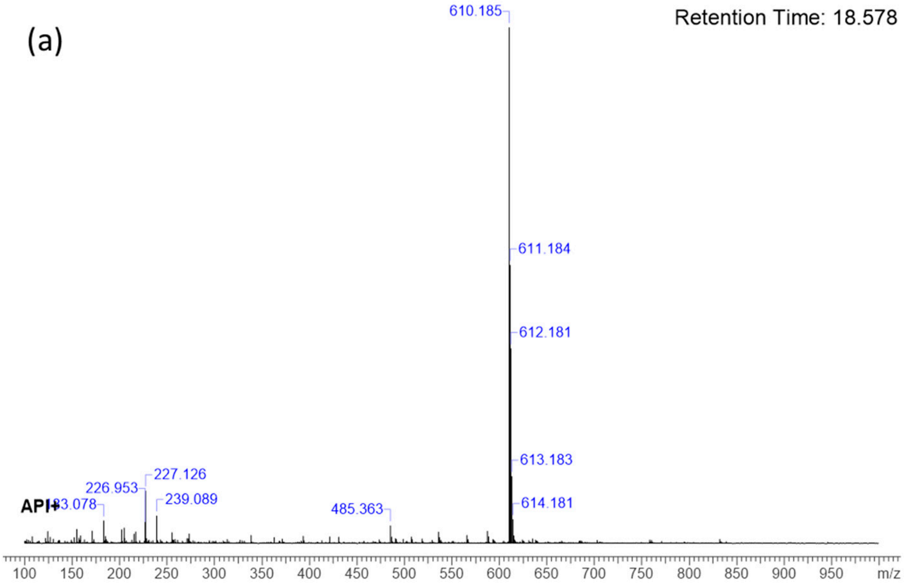

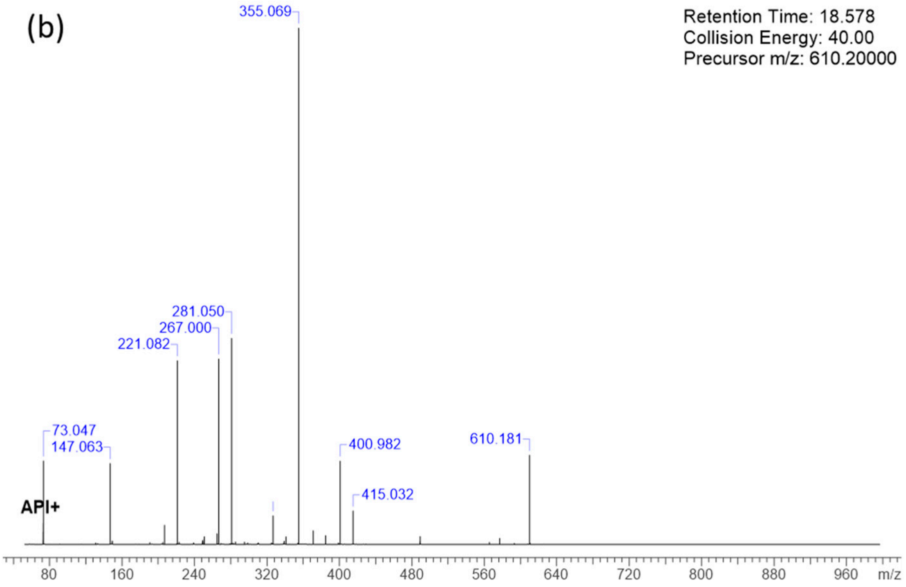

Figure S7. UHPLC-ESI(+)–HRMS peak of compound 7, identified as anthocyanidin glycoside (peonidin 3-rutinoside or peonidin 3-rhamnoside 5-glucoside): (**a**) full-scan MS; (**b**) MS² spectrum.

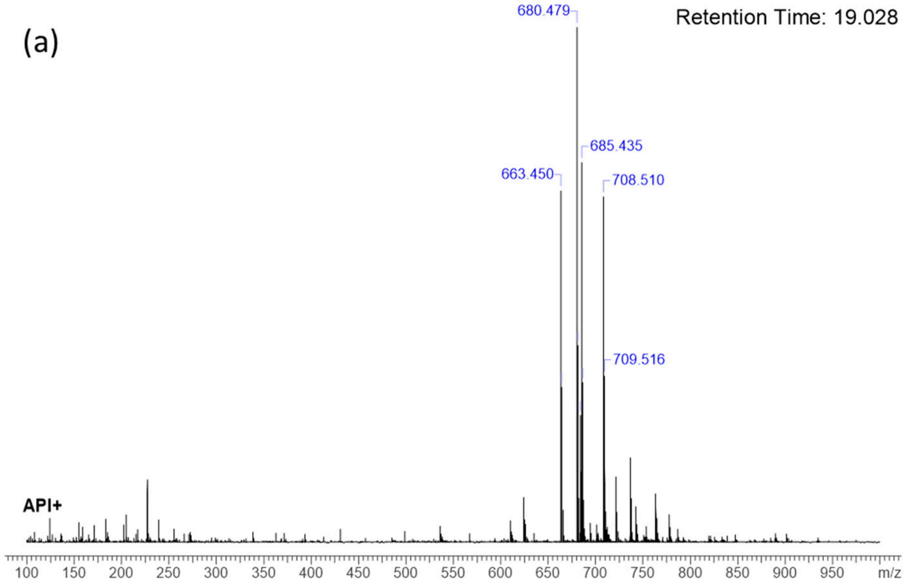

(b)

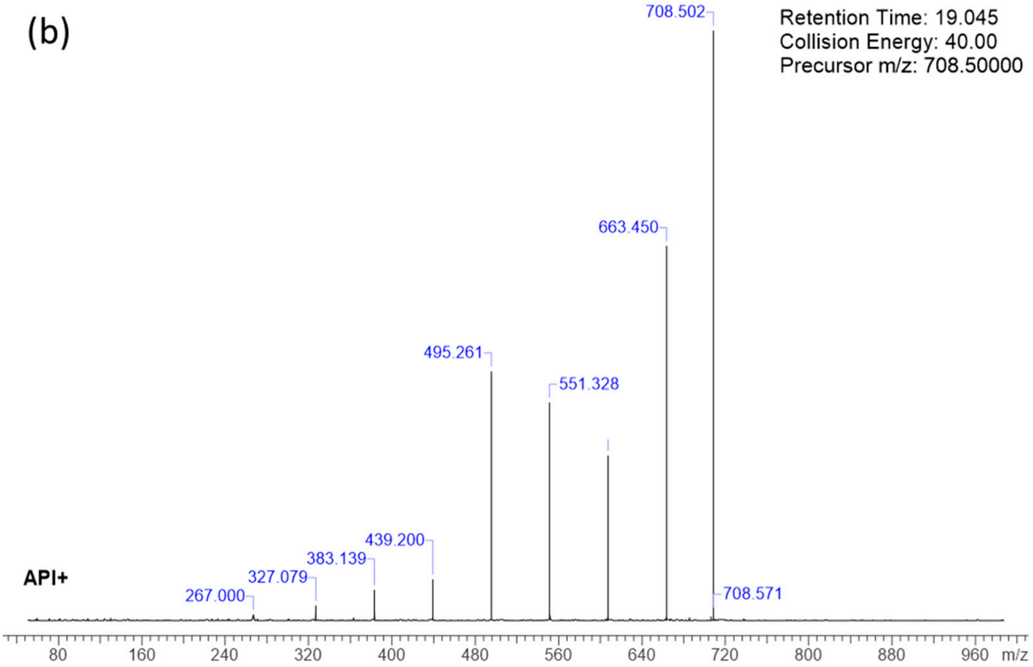

Figure S8. UHPLC-ESI(+)–HRMS peak of compound 8, identified as phosphoserine (PS(O-18:0/13:0) or PS(O-16:0/15:0): (**a**) full-scan MS; (**b**) MS^2 spectrum.
